# Transcription Factors, R-Loops and Deubiquitinating Enzymes: Emerging Targets in Myelodysplastic Syndromes and Acute Myeloid Leukemia

**DOI:** 10.3390/cancers13153753

**Published:** 2021-07-26

**Authors:** Silvia M. L. Barabino, Elisabetta Citterio, Antonella Ellena Ronchi

**Affiliations:** Department of Biotechnology and Biosciences, University of Milano-Bicocca, 20126 Milan, Italy; silvia.barabino@unimib.it

**Keywords:** myelodysplastic syndromes (MDS), acute myeloid leukemia (AML), transcription factors, RNA splicing, R-loops, genome integrity, deubiquitinating enzymes (DUBs)

## Abstract

**Simple Summary:**

The advent of DNA massive sequencing technologies has allowed for the first time an extensive look into the heterogeneous spectrum of genes and mutations underpinning myelodysplastic syndromes (MDSs) and acute myeloid leukemia (AML). In this review, we wish to explore the most recent advances and the rationale for the potential therapeutic interest of three main actors in myelo-leukemic transformation: transcription factors that govern myeloid differentiation; RNA splicing factors, which ensure proper mRNA maturation and whose mutations increase R-loops formation; and deubiquitinating enzymes, which contribute to genome stability in hematopoietic stem cells (HSCs).

**Abstract:**

Myeloid neoplasms encompass a very heterogeneous family of diseases characterized by the failure of the molecular mechanisms that ensure a balanced equilibrium between hematopoietic stem cells (HSCs) self-renewal and the proper production of differentiated cells. The origin of the driver mutations leading to preleukemia can be traced back to HSC/progenitor cells. Many properties typical to normal HSCs are exploited by leukemic stem cells (LSCs) to their advantage, leading to the emergence of a clonal population that can eventually progress to leukemia with variable latency and evolution. In fact, different subclones might in turn develop from the original malignant clone through accumulation of additional mutations, increasing their competitive fitness. This process ultimately leads to a complex cancer architecture where a mosaic of cellular clones—each carrying a unique set of mutations—coexists. The repertoire of genes whose mutations contribute to the progression toward leukemogenesis is broad. It encompasses genes involved in different cellular processes, including transcriptional regulation, epigenetics (DNA and histones modifications), DNA damage signaling and repair, chromosome segregation and replication (cohesin complex), RNA splicing, and signal transduction. Among these many players, transcription factors, RNA splicing proteins, and deubiquitinating enzymes are emerging as potential targets for therapeutic intervention.

## 1. Introduction

Hematopoietic stem cells (HSCs) ensure continuous production of all blood cell types throughout life. By means of a delicate equilibrium between self-renewal, quiescence, and differentiation, HSCs maintain homeostatic conditions and dynamically respond to stress stimuli. The balanced production of the different cell lineages physiologically varies during aging, when the hematopoietic potential is progressively skewed towards the myeloid lineages at the expenses of immune cells [1,2,3]. The accumulation of heritable genetic mutations in individual cells and the kinetics of their selection can lead to myeloid neoplasms, a heterogeneous group of diseases characterized by the dysfunctional production of myeloid cells in the bone marrow. This can manifest in cytopenia and cellular dysplasia, such as in myelodysplastic syndromes (MDSs), in the overproduction of mature clonal myeloid elements, such as in myeloproliferative neoplasms (MPN), or both, as it happens in MDS/MPN, which share different molecular and clinical traits and present both myelodysplastic and proliferative features [4]. Myeloid neoplasms entail a high risk of developing into acute myeloid leukemia (secondary AML or s-AML). AML can also develop as a late complication in patients after leukemogenic therapies (therapy-related AML or t-AML), or without clinical history of prior MDS or known exposure to potentially leukemogenic agents (de novo AML) [5]. Driver mutations leading to a preleukemia condition originate in HSC or in hematopoietic stem-progenitor cells (HSPCs). The preleukemic clones can have a variable latency and, in some cases, can persist for years before further mutations trigger their leukemic evolution [4,6].

MDS has an estimated crude incidence of 4 to 5 cases per 100,000 persons per year. Although MDS occurs at all ages, the incidence is higher in elderly individuals, with a median age of 70 years old at diagnosis [4]. Careful evaluation of the individual patient prognostic risk, genetics, and age guides the clinical decision-making process [4]. Although current drugs can temporarily modulate myelodysplastic hematopoiesis, they fail in eradicating the disease [4,7].

Failure of current therapies in eradicating MDS/AML is attributed to the persistence of leukemic stem cells (LSCs) upon treatment and to the emergence of resistant subclones [8]. Notably, recent deep-sequencing studies revealed the possibility that relapse from chemotherapy can occur not only from LSCs that are resistant to the treatment but also from pre-leukemic mutated but not transformed HSCs, which are already present in the patient at diagnosis or during clinical remission, and which can acquire additional mutations, further highlighting the complexity and heterogeneity of the disease [9,10]. LSCs share functional properties with normal HSCs [11]. Indeed, LSCs are functionally defined as cells capable of self-renewal and of propagating the disease upon transplantation into immunodeficient mice [8]. In addition to self-renewal capacity, LSCs co-opt many survival mechanisms typical to HSCs to their advantage, including genome maintenance processes, epigenetic and stemness transcriptional programs, the pre-mRNA splicing machinery, metabolic properties, interaction with the microenvironment, and inflammatory signals [11,12]. In fact, recurring mutations in regulators of gene expression, including epigenetic proteins, transcription factors (TFs), and the components of the splicing machinery, are found in 70% of AML patients [13,14]. Understanding the molecular mechanisms regulating HSC biology and their dysregulation in pre-leukemic HSCs and in LCSs is therefore critical for understanding the disease and for developing therapies that harness cancer HSPC-specific vulnerabilities and ultimately eradicate the disease.

## 2. Genetics Underlying Evolutionary Trajectories of MDS Progression

**Figure 1:**
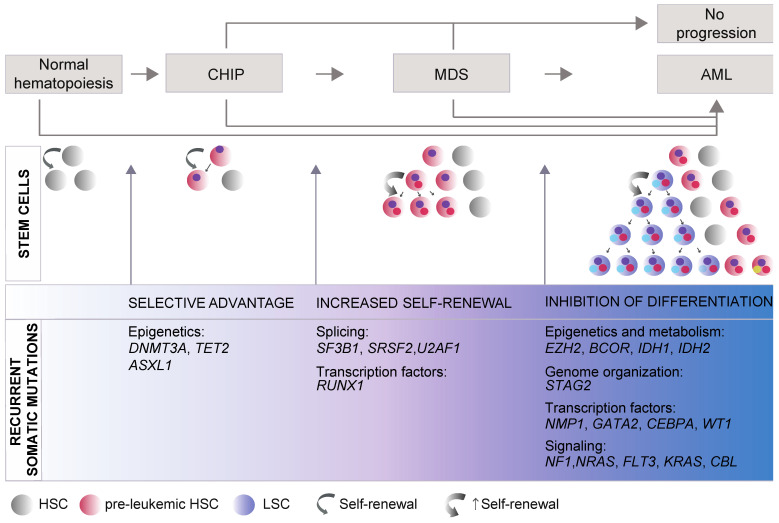
General model of myelodysplastic syndromes (MDS) progression. MDS is a progressive disease, developing through the accumulation of mutations in hematopoietic stem cells (HSCs) or early progenitors that promotes the growth and spread of somatically mutated clones in the bone marrow (BM) and eventually leads to overt clinical disease and to secondary acute myeloid leukemia (s-AML). Distinct phases can be recognized. During the initial phase of clonal hematopoiesis (CH), an initiating driver mutation provides selective advantage and promotes expansion of mutant single clones, without morphological evidence of malignancy nor cytopenia. When the cells carrying the somatic mutation reach 4% of all BM cells (corresponding to a variant allele frequency of at least 2% for the mutation), this condition is defined as CH of indeterminate potential (CHIP). Mutations in the genes involved in DNA methylation, *DNMT3A* and *TET2*, are the most frequent genetic lesions in CHIP followed by mutations in *ASXL1*, *JAK2*, *SF3B1*, and *TP53* [16,17,18,19]. In the second phase, clonal hematopoiesis progressively expands and becomes dominant in the BM. Accrual of genetic lesions that promote self-renewal/proliferation and inhibit progenitor’s differentiation leads to clonal expansion, dysplasia of progenitors, and mature cells cytopenia. Patients who acquire splicing factor mutations (*SF3B1*, *SRSF2*, and *U2AF1*) are at higher risk of developing overt dysplasia and MDS. MDS is clinically defined by a variant allele frequency of the founding mutation of at least 20% of BM cells [4,20]. The leukemic phase (s-AML) is characterized by the acquisition of mutations that inhibit differentiation and typically drive clonal selection and transformation of pre-leukemic HSCs into leukemic stem cells (LSCs), which produce leukemic blasts with aggressive proliferation and expansion abilities. The diagnosis of s-AML is made when the proportion of blast cells increases to 20% or more [4]. Although there is a clear stepwise progression from CHIP to MDS and to s-AML, MDS and AML can also occur due to de novo mutations and CHIP can evolve directly in AML or, in most of the cases, never progress [21,22]. The most frequently mutated genes driving each dysregulated phase are listed and are derived from the following sources: CHIP [16,17,18,19,23], MDS [4,13,24], and s-AML [5,25]. Recently, high-coverage whole genome sequence studies of large cohorts of MDS patients identified only 1 driver mutation in 90% of individuals with CHIP [19], progressing to a median number of two to three driver mutations per patient at the onset of the MDS/sAML [25]. While the initial driver mutation occurs in a hematopoietic stem cell capable of self-renewal, mutations associated with clonal expansion during disease progression may occur in progenitor cells, endowing them with self-renewal properties [26,27]. HSC: hematopoietic stem cells; LSC: leukemic stem cells.

MDS entails both molecular and cytogenetic complexity. Early studies and more recent analysis through whole-genome sequencing of somatic mutations in MDS patients identified more than 50 recurrently mutated genes. These genes can be broadly divided into a few main categories controlling basic cellular functions: (1) transcriptional and epigenetic control of gene expression; (2) RNA splicing; (3) genome integrity; and (4) signal transduction pathways. Notably, only six genes are most frequently mutated and found in at least 10% of patients with MDS: *DNMT3A*, *TET2*, *ASXL1*, *SF3B1*, *SRSF2*, and *RUNX1* [13,15,16]. These studies also pointed to major mutation-driver genes acquired in each of the phases during the course of the disease, from initial clonal hematopoiesis (CH), characterized by the presence of HSC/hematopoietic precursors carrying somatic mutations, through to development of MDS and eventually progression to AML (Figure 1), suggesting that CH–MDS–AML development constitutes a continuous evolutionary process.

### 2.1. Somatic Mutations

Mutations in the epigenetic regulators *DNMT3A*, *TET2*, and *ASXL1* have been found in HSCs or in multipotent progenitors [28,29] in most CH cases (also termed CH of indeterminate potential, CHIP, or age-related CH) [16,18,19]. Individuals with CHIP are often elderly and apparently healthy individuals with normal blood counts [19]. However, CHIP has an estimated annual risk of approximately 0.5–1% of developing into hematologic malignancy with decreased survival [18]. CHIP can be transferred from donor to recipient during allogenic hematopoietic stem cell transplantation (alloHSCT) [30]. For this reason, the eligibility of stem-cell donors with CHIP should be evaluated with caution. While emerging evidence supports an impact of CHIP on the incidence of donor cell leukemia (DCL), the associated long-term risk of evolution to MDS-AML is still poorly defined [30,31,32].

*TET2* and *DNMT3A* are the most commonly mutated genes not only in CHIP but also in (early) MDS, suggesting a role in the early development of MDS as founder mutations [16]. During the transition to MDS, spliceosome mutations (*SF3B1*, *SRSF2*, and *U2AF1*) become predominant early events. Moreover, individuals who acquire splicing mutations show a more rapid progression to overt dysplasia, consistently with a role of these genes in driving clonal dominance [4,11,18]. Mutations in transcription factors (runt-related transcription factor 1 (*RUNX1*), GATA-binding protein 2 (*GATA2*), cut-like homeobox 1 (*CUX1*), and CCAAT enhancer-binding protein alpha (*CEBPa*) are also typical in MDS; however, they can occur either early or later in the progression of the disease [13].

Progression to secondary AML (s-AML) is associated with clonal expansion or emergence of a subclone from either pre-leukemic HSCs or their progeny of pre-leukemic progenitors with a unique set of mutations. Different clones can also coexist in the patient and parallel clonal evolution at the level of HSCs can occur and account for disease progression, as single-cell studies recently demonstrated [27,33,34]. The acquired mutations typically abrogate differentiation and, under conditions of selective pressure, this event drives leukemogenesis. Mutations in the genes *EZH2*, *BCOR*, *TP53*, or *STAG2* are frequently found in s-AML and together with the splicing genes *SRSF2*, *SF3B1*, *U2AF1*, and *ZRSR2*, they are highly specific for s-AML compared to de novo AML, reflecting its evolution from MDS [5]. In addition, mutations in genes involved in signal transduction, including *FLT3*, *NRAS*, and *PTPN11*, as well as mutations in the transcription factors *NPM1* and *WT1*, and in the metabolic enzymes *IDH1* and *IDH2*, tend to be newly acquired during progression to s-AML and are associated with a higher risk of s-AML and shorter survival [25]. The tumor-suppressor TP53 protein is recurrently mutated in MDS, with mutations present in 5% of the patients and increasing to 10% of patients with AML [16] and to 50% in patients exposed to chemotherapy, often associated with a complex karyotype, disease progression, and poor prognosis [16,35,36].

Through progression from CHIP to MDS/AML, co-occurrence or mutual exclusivity between gene and/or chromosome alterations are often found and support functional interactions involved in positive or negative selection of the mutated hematopoietic clone carrying a given set of mutations [15,16,22]. The evaluation of these antagonistic/synergistic effects has a critical clinical role as a prognostic factor and for its therapeutic implications [14,16,37].

### 2.2. Familial Syndromes Predisposing to MDS

Although most cases of MDS are caused by somatic genetic lesions, there are several rare familial syndromes in which inherited mutations predispose to bone marrow failure and to early onset of MDS [4]. These include diseases related to mutations in the DNA damage response (DDR) and in the DNA repair pathways, such as Fanconi anemia [38] and ERCC6L2-associated bone marrow failure syndrome [39]; telomere maintenance, such as in dyskeratosis congenita or telomeropathies [40,41]; TP53 loss-of-function in Li-Fraumeni syndrome [42]; and two diseases due to inherited GATA-binding protein 2 (GATA2) mutations, MonoMAC syndrome [43] and Emberger syndrome [44].

## 3. Transcription Factors in MDS and AML

The main characteristic of preleukemic and leukemic cells is the failure to complete their terminal differentiation program. Unsurprisingly, transcription factors (TFs) controlling the equilibrium between proliferation, differentiation, and apoptosis at the end point of the signaling pathways are often involved in preleukemic and leukemic transformation in MPN, MDS, and AML, as well as in other forms of cancer [45]. Cell fate specification by TFs relies on common mechanisms, including the activation of subsets of lineage-specific genes, the inhibition of alternative lineages, the block of cell proliferation, and the induction of apoptosis. In some cases, overexpression or ablation of key TFs results in cell fate changes [46]. Once established, lineage choice is often reinforced by autoregulatory circuits [47,48].

In hematopoiesis, TFs, such as RUNX1, TAL1, GATA2, GFI, and MYB, are important for HSPC maintenance and their inactivation affects multiple blood lineages. Instead, other factors, such as PU.1, CEBPa, KLF1, GATA1, NFE2, GFI1B, and SOX6, are required for the proper differentiation of a more restricted number of lineages or of single lineages. Often, the same factor can play roles in both HSCs and the more committed precursors [49].

Mutations in TFs are found both in MDS/AML somatic cells and in the germline, where they predispose to secondary leukemia [50]. A variety of molecular mechanisms underlie TFs’ oncogenic mutations: genomic rearrangements (translocations, deletions, and inversions), genic mutations, regulatory mutations, and mutations leading to the selective expression of specific TF isoforms. This heterogeneity reflects upon different functional outcomes: gain of function, loss of function, or dominant negative effects, as exemplified by the case of RUNX1, discussed here below.

In this review, we do not aim to provide a full list of the TFs involved in myeloid transformation but only to illustrate the above mechanisms focusing on representative examples.

### 3.1. Genome Rearrangements Involving TFs

Large chromosomal rearrangements are common in MDS and are identified by conventional karyotyping of bone marrow cells in 50–60% of the patients [51]. Most of these rearrangements are unbalanced changes, resulting in chromosomal loss or gain. Common deletions include deletion of chromosome 5q [52,53], loss of chromosome 7 or of the region 7q (containing *CUX1* [54], and deletion of 17p (including p53) and of 20q [16,55]. Other frequent rearrangements in AML are associated with specific chromosomal translocations that result in the formation of chimeric proteins, often involving transcription factors [56]. Translocations typically alter the binding properties of the fusion protein and define the phenotype of the resulting myeloid defect [16,55]. The most common translocations found in AML involving TFs are *AML1*-*ETO*, *CBFB*-*MYH11*, *PML*-*RARa*, *MLL*-*AF9*, *MML*-*ENL*, and *FUS*-*ERG*. Of these, here we will discuss the t(8;21) *AML1*/*ETO* (*RUNX1*/*RUNX1T1*) and translocations involving FET proteins, such as the *FUS*/*ERG* fusion, as examples of the complex outcome of the translocation event.

RUNX1, also known as AML1, CBFa2, or PEBP2aB, is a transcription factor essential for definitive hematopoiesis, belonging to the core-binding factor (CBF) family [57,58,59]. RUNX1 can directly bind to DNA but its affinity for DNA is greatly increased by the presence of its heterodimeric partner, the core binding factor-β (CBFβ), which is also rearranged in some AML by inversions and translocations [60]. The major consequence of the *AML1/ETO* fusion is that the ETO (RUNX1T1) moiety, fused to the DNA-binding domain of RUNX1, recruits the corepressors N-CoR/mSin3/HDAC1 [61]. As a result, the AML1/ETO fusion protein competes with RUNX1 for the same DNA-binding sites, acting as a dominant repressor of the RUNX1 targets that control the activation of the signal transduction pathways, proliferation, and apoptosis. In addition, AML1/ETO inhibits the transcription of *PU.1*, *GATA1*, and *CEBPa*, blocking the later stages of myeloid differentiation [62,63,64].

Recent experiments, mapping by Precision RUN-ON sequencing after chemical-induced degradation of AML1/ETO, revealed that the very early targets are very few and include critical myeloid differentiation genes, such as *CEBPa*, *PLZF* (*ZBTB16*), *NFE2*, *MTG16* (*ETO2*/*CBFA2T3*), and *GFI1B* [65].

The *AML1*/*ETO* fusion RNA contains the *ETO* 3’UTR that has been suggested to act as a miRNA sponge, contributing to the widespread gene expression deregulation of cancer cells [66]. Finally, AML1/ETO alters the alternative splicing process by deregulating the expression of splicing factors and influences the selection of the transcription start sites, possibly contributing to the frequent use of alternative promoters observed in cancer cells [67].

The availability of large sets of sequencing data on *AML*/*ETO*-expressing cells uncovered numerous *AML*/*ETO* co-occurring mutations, which can influence AML prognosis and relapse. These events comprise both additional chromosomal alterations and gene mutations affecting tyrosine kinases (including *KIT*, *JAK2*, *FLT3*, *NRAS/KRAS*, *CBL*, and *PTPN11*) and MYC signaling, members of the cohesin complex (*RAD21*, *STAG2*, *SMC1a*, and *SMC3*), or epigenetic (i.e., *EZH2*, *KDM6A*, and *TET2 ASXL1*/*2*) and transcriptional regulators, together with other less represented genes [68,69,70,71].

Translocations involving *FUS* (fused in liposarcoma), *EWSR1* (Ewing sarcoma RNA-binding protein 1), and *TAF15* (TATA-binding associated factor 15), the three members of the FET family of RNA-binding proteins originally identified in sarcoma [72], are also present in AML [73,74,75,76]. FET proteins are DNA/RNA-binding proteins that share a common domain structure with a low complexity (LC) domain enriched in serine, tyrosine, glutamine, and proline residues at the N-terminus; a central DNA/RNA-binding domain; and a C-terminal nuclear localization signal. Translocations give rise to in-frame fusion proteins comprising the N-terminal part of a FET protein and the DNA-binding domain of a transcription factor, which often belongs to the ETS family at the C-terminus [77].

Although the oncogenic phenotype has been replicated in Mx1-cre mice where the Cre-inducible expression of the *EWSR1*/*FLI1* fusion in hematopoietic cells results in the rapid development of myeloid/erythroid leukemia [78], the molecular mechanisms of transformation by FET fusions are still not completely understood. The FET LC domain mediates interaction with RNAPII and TFs ([79] and references therein), suggesting a direct role in transcriptional regulation and/or transcription-coupled RNA splicing. Indeed, fusion proteins were reported to act as transcriptional activators that bind to the same DNA sequences as the wild-type transcription factors and rely on the presence of the FET LC domain-mediated transactivation. Consistent with a role of the fusion proteins in transcription regulation, Sotoca and colleagues recently showed that the FUS/ERG fusion controls the expression of genes involved in the maintenance of the HSC phenotype [80]. However, there is also evidence that the expression of FET proteins translocations may induce oncogenic transformation through pathways that are independent of the TFs’ DNA-binding domain moieties [81,82]. Since FUS, EWSR1, and TAF15 participate in RNA splicing [83,84], one possible pathogenic mechanism could involve aberrant mRNA synthesis and the accumulation of RNA/DNA hybrids (R-loops), as described below, in Section 5.1.1. Another potentially relevant phenomenon that could be perturbed by FET fusion proteins is liquid–liquid phase separation (LLPS). LLPS is a phenomenon in which solutions of proteins condense into a dense phase that often resembles liquid droplets [85]. The assembly of protein condensates through LLPS plays crucial roles in many biological processes, including DNA repair and transcription [86]. The N-terminal LC domains of FUS and EWSR1 have been recently shown to mediate protein multimerization and physiological LLPS [87,88,89]. Thus, dysfunctional LLPS could explain, at least in part, the oncogenic properties of translocations involving FET proteins. Consistent with this idea, the EWSR1/FLI1 fusion protein undergoes LLPS in vitro, suggesting that its ability to drive transcriptional programs that lead to cancer may, at least in part, depend on this property [87].

In addition to transcription and splicing, the FET proteins participate in the maintenance of genomic stability [79]. We have recently provided evidence that FUS drives the assembly of DNA repair condensates at DNA double-strand break sites [90]. Thus, on the one hand the translocated FET proteins’ LC domains could disrupt the assembly of functional DNA repair condensates, contributing to the genomic instability observed in leukemic cells. On the other hand, they could promote the assembly of aberrant condensates at enhancers and promoters, thus driving abnormal tumorigenic transcriptional programs. Understanding if all FET fusion proteins perturb protein condensates would be helpful in designing novel therapeutic approaches.

### 3.2. Gene Mutations and Allelic Variants

The increasing number of MDS/AML gene expression profiling and genome sequencing has revealed an extremely complex pattern of mutations of the genes involved [15]. These mutations can alter the structure of the protein and/or its level of expression. The outcome can either change the transcriptional/epigenetic activity of the protein and/or its protein–protein interactions, with the subsequent alteration in the regulatory circuits. This type of mutation will define the characteristic of the MDS/AML clone, including the different outcome in terms of prognosis and response to treatment.

For example, the most common *RUNX1* mutations are missense, nonsense, and frameshift mutations, affecting the DNA-binding domain or the heterodimerization domain, resulting in a dominant negative effect (https://runx1db.runx1-fpd.org, accessed on 14 July 2021). Missense mutations primarily hit the RUNT domain and affect DNA binding, whereas frameshift and nonsense mutations fall throughout the protein [50]. Of interest, in MDS/AML, *RUNX1* is often found mutated in combination with *SRSF2*, *ASXL1*, and *STAG2*, this latter being part of the cohesin complex [16,91]. In particular, RUNX1 colocalizes with STAG2 on active enhancers. The synergistic effect of the double *Stag2* and *Runx1* KO in mice suggest that RUNX1 contributes to the maintenance of the proper enhancer-promoter chromatin looping in HPSCs cells [92].

Allelic variants can also predispose to myeloid neoplasms, as in the case of *GFI1*. *GFI1* is expressed in HSCs, as well as in lymphoid and myeloid precursors, where it regulates important myeloid genes, such as *HOXA9*, *PBX1*, *MEIS1*, *CSF1*, and *CSFR1* [93]. The two allelic GFI variants differ for an amino acid at position 26: the most common allele carries a serine (GFI26S), whereas that rarer allele carries an asparagine (GFI126N) and is associated with both MSD and AML [94]. The knock-in in mice of the two different human allelic variants demonstrated that GFI126N promotes the proliferation of myeloid precursors. Moreover, data from both mice and patients’ cells demonstrated that GFI126N compromises the epigenetic status of the GFI target genes, causing their partial de-repression [95]. Beside its transcriptional repressor function, GFI has non-canonical activities related to DNA repair genes: it promotes PRMT1 recruitment and subsequent methylation of MRE11 and 53BP1, which is necessary for their function in the DNA damage response [96]. Low levels/loss of GFI both in the mouse [97] and humans [98] are associated with the accumulation of myeloid precursors, predisposing to AML, as it also happens in the deletion of the 1p region, containing the *GFI* gene [99]. The restoration of GFI levels resumes differentiation [100]. Of interest, in mice, the reduced expression, but not the complete ablation, causes a fatal myeloproliferative neoplasm [97].

Finally, GFI1B, the GFI ortholog, is essential not only for HSC [94] but also for the erythroid and megakaryocytic lineages [101,102]. *GFI1B* expression is reduced in MDS/AML patients and loss of *GFI1B* alleles in mouse models accelerates AML [103]. Beside these dosage effects, a dominant somatic *GFI1B* mutation has been identified in AML [104].

### 3.3. Gene Dosage Effects

As already mentioned before, because of their ability to control several downstream genes acting in a combinatorial manner, even subtle changes in the quantity of TFs can have a notable impact. This implies that mutations altering the expression levels of key TFs driving lineage commitments and differentiation can be relevant. In the case of myeloid cancers, this principle is illustrated by a variety of examples, including both TFs expressed in HSPC and those specifying the identity of more differentiated cells.

Mutations causing RUNX1 deficiency are detrimental for myeloid differentiation: heterozygous germline mutations result in a highly penetrant familial platelet disorder with a predisposition to AML [105] and somatic heterozygous *RUNX1* mutations are recurrent in myelodysplastic syndromes and in AML with adverse outcome [106]. In the 30% of cases, *RUNX1* mutations occur on both alleles or are associated with loss of heterozygosity, leading to a complete RUNX1 loss. The outcome of these mutations indicates that the level of RUNX1 is critical for leukemia [107].

Like RUNX1, GATA2 is crucial for the production and maintenance of HSCs in embryonic and adult hematopoiesis and HSCs are very sensitive to the GATA2 levels [108]. As a consequence, qualitative or quantitative mutations impairing *GATA2* expression are recurrent in several hematopoietic diseases, including MDS and AML [109]. One of the better-described examples of a “regulatory” mutation affecting the level of a TF in AML is the 3q21;q26 inversion. By changing the position of the *GATA2* myeloid distal enhancer, this rearrangement simultaneously causes a functional downregulation of *GATA2* in myeloid precursors and the overexpression of the proto-oncogene *EVI1*, whose overexpression in HSCs is associated to MDS and AML [110,111,112]. More recently, Kozyra and colleagues [113] reported that heterozygous synonymous mutations of *GATA2* can cause loss of the mutant mRNA, without altering the protein function. This new type of mutation adds to the already-known causes of GATA2 deficiency predisposing to MDS/AML [113].

### 3.4. TFs Cross-Antagonism: The Paradigmatic Example of PU.I and GATA1

PU.1 and GATA1 play an antagonistic role in directing differentiation toward a myeloid (granulocytes and macrophages) versus the erythro/megakaryocytic lineages: cells primed toward the myeloid cell fate express *PU.1* and repress *GATA1*, whereas the opposite occurs in cells that will differentiate into the erythroid lineage [114]. The direct cross-inhibition between GATA1 and PU.1 is reciprocal: PU1 blocks GATA1 transcription [115] and protein [116] whereas GATA1, as well as GATA2, inhibit PU.1 [115].

PU.1 regulates almost every myeloid gene, including the receptors for granulocyte–macrophage colony-stimulating factor (GM–CSF), macrophage colony-stimulating factor (M–CSF), and granulocyte colony-stimulating factor (G–CSF) [117], whereas virtually all erythroid genes contain GATA1-binding sites in their regulatory regions [118]. Of interest, once PU.1 has primed cells toward the myeloid lineage, CEBPa controls uniquely the expression of the G–CSF receptor, an indication of its more restricted role in the specification of the granulocyte lineage. This would also explain the antagonism at later stages between CEBPa—required for granulocytic differentiation—and PU.1, essential for monocyte/macrophage differentiation [119,120].

The generation of mouse models expressing different levels of PU.1 clearly demonstrated the PU.1 dosage-dependent development of AML. In fact, PU.1 halpoinsufficiency (50% of the protein) is not sufficient to induce leukemia, whereas hypomorphic alleles producing 20% of the normal level causes AML because of the accumulation of abnormal precursor blasts, which frequently undergo further chromosomal rearrangements. These AML blasts resume differentiation upon *PU.1* exogenous expression [121]. The complete *PU.1* ablation is lethal due to a multilineage myeloid-lymphoid defect [122].

*PU.1* expression is under the control of a myeloid enhancer [47,123]. In humans, a particular SNP within this element, which decreases enhancer activity, is frequent in AML with a complex karyotype. The minimal reduction in PU.1, artificially obtained through the heterozygous enhancer mutation in mice, results in AML progression when carried in combination with *Msh2* deficiency, a defect which impairs DNA mismatch repair. This suggests that a modest PU.1 reduction can act as driver mutation [124]. Interestingly, the reduction in PU1 and CEBPa levels caused by aberrant ubiquitination signals have been recently described in AML, further demonstrating the importance of a proper TF protein dosage [125,126].

In normal hematopoiesis, *GATA1* [118] starts to be expressed in early progenitors; it increases prior to the proerythroblast stage and then declines during terminal maturation. *Gata1* knock-out in mice leads to embryonic death due to severe anemia [127]. Instead, megakaryoblasts lacking GATA1 undergo abnormal proliferation and fail to terminally differentiate [128]. The generation of mouse models expressing different levels of GATA1 demonstrate that GATA1 amounts must be tightly regulated to balance proliferation and differentiation. *Gata1* is on the X chromosome: male mice expressing 5% of the protein (*Gata1.^05^* strain [129]) die around day E12.5 because of anemia, as *Gata1* null mutants do, whereas *Gata1.^05^* females frequently develop erythroblastic leukemias. In contrast, heterozygous *Gata1*-null female mice do not develop. A reduction in GATA1 to 20% (*Gata1*^low^ model [128]) is sufficient to sustain adult (but not primitive) erythropoiesis. However, *Gata1*^low^ mice present severe thrombocytopenia associated with the accumulation of megakaryocytic progenitors. This phenotype resembles primary myelofibrosis (PMF), where the GATA1 reduction is secondary to deficiency in RPS14 and causes a clonal malignancy predisposing to AML [130]. On the other hand, the artificial overexpression of GATA1 in mouse erythroid cells induces cell cycle arrest and apoptosis at the proerythroblast stage [131]. In humans, *GATA1* overexpression is observed in essential thrombocythemia and in polycythemia vera, diseases consisting of the overproduction of platelet and red blood cells, respectively [132].

### 3.5. Mutations Leading to the Selective Expression of Specific TFs Isoforms

GATA1 presents two protein isoforms: full length GATA1 (GATA1-FL) and a shorter GATA1 isoform (GATA1s), which is absent in adults and lacks the N-terminal activation domain (N-TAD). GATA1s is generated by N-terminal mutations causing the translation from an ATG codon located in the third exon [133]. The rare inherited *GATA1* mutations resulting in predominant *GATA1s* expression cause Diamond–Blackfan Anemia, a hypoplastic anemia syndrome [134].

Acquired mutations during fetal liver erythropoiesis leading to *GATA1s* expression are involved in myeloid leukemia of down syndrome (ML-DS)—an acute leukemia with megakaryoblastic and erythroid traits that evolves from a preleukemic clonal condition (transient abnormal myelopoiesis, TAM) present in about 30% of trisomy 21 (T21) patients. TAM spontaneously regresses in the majority of cases but in about 10% of them it evolves in leukemia [135]. The region of chromosome 21 increasing the risk of leukemia contains genes involved in hematopoiesis, such as *RUNX1*, *ETS2*, *ERG*, and *DYRK1A* [136]. However, the molecular mechanisms of T21 contribution to fetal hematopoiesis is still unclear. Indeed, the comparison between TAM and ML-DS samples demonstrated that TAM requires both trisomy 21 and the *GATA1s*-associated mutation. Further progression to ML-DS requires additional cooperative mutations [137].

CEBPa is a transcription factor essential for myeloid differentiation mutated in about 10% of AML cases. Although considered a myeloid factor, its knock-out in HSCs, where it is expressed at a low level, is associated with HSC expansion [138,139].

CEBPa is a bzip protein coded by an intron-less gene containing a zipper domain required for dimerization, a basic region responsible for DNA binding, and two transactivation domains at the N terminus [138,139]. The *CEBPa* gene’s alternative start codon usage generates a full-length protein of 42 kDa (p42), a shorter version of 30 kDa (p30) lacking the full trans-activation domain [140], and a less characterized extended protein translated from a non-AUG initiating codon [141]. The p42/p30 ratio is regulated at the translational level by eIF2a/eIF3E, which promotes p30 translation under pro-proliferative conditions [142].

Recurrent *CEBPa* AML-associated mutations are nonsense or frameshift mutations resulting in the exclusive expression of the p30 isoform from the hit allele. Because the p30 isoform maintains the dimerization and DNA-binding domains, it acts as a dominant negative, blocking the transcriptional activation of the genes driving myeloid lineage specification downstream to CEBPa [138,139]. A second class of *CEBPa* mutations falls in its DNA-binding or heterodimerization domain. Biallelic mutants often carry these two types of mutations on the two different alleles, with the result that only the p30/p30 dimers are able to bind DNA.

A frequent co-mutation in biallelic *CEBPa* AML is represented by heterozygous *GATA2* mutations in Zinc Finger 1 (ZnF1) [143,144,145], which are thought to reduce p30/p30 transcriptional activation [143]. More recently, allele-specific expression (ASE) of *GATA2* due to promoter methylation has been reported in the *CEBPa* biallelic-mutant AML. In cases carrying *GATA2* mutations, the mutated allele is preferentially expressed, suggesting that ASE can cooperate with the *GATA2* mutations [146].

The co-occurrence of *GATA2* and *CEBPa* biallelic mutations is statistically significant in bi-lineage acute erythroid leukemia (AEL) [147]. In AEL, mutated CEBPa increases the erythroid-specific lineage TFs; the *GATA2-ZnF1* mutation induces erythroid TFs and reduces chromatin accessibility to myeloid TFs. The ability of GATA2 to interact with both myeloid and erythroid TFs is likely the key to control their differential chromatin accessibility [148].

### 3.6. The Heterogeneous Spectrum of TFs and Mutation Involved in MDS/AML

Although the TFs (and their mutations) described above are the ones with a clearer involvement in MDS/AML, the increasing number of experimental data from patients’ cells and experimental models clearly points to the involvement of additional TFs in MDS/AML genesis.

For example, KLF1 [149], like GATA1, is critical for many aspects of erythropoiesis, including the regulation of globins genes expression. Recently, it has been shown that AML cells express high levels of KLF1 that binds to genes commonly altered in MDS/AML, such as *NPM1*, *SF3B1*, *KDM6A*, and *CREBBP* [150].

NFE2 is a transcription factor mainly expressed in erythroid, megakaryocytic, and mast cells [151]. Although *Nfe2*-deficient mice only present moderate defects in megakaryocyte formation, its overexpression recapitulates in mice the phenotype of MNP, with expansion of the HSPCs compartment and a predisposition to AML, which occurs with the acquisition of secondary mutations, such as rearrangements. Accordingly, in humans, NFE2 has been found mutated in MPN patients [152].

A key question in defining the role of TFs in cancer development is to what extent cell cycle disruption, differentiation, and apoptosis are intertwined. We recently studied this aspect by analyzing the role of SOX6 in erythropoiesis, where it is required for terminal differentiation of erythroid cells under normal and stress conditions [153,154]. In AML, SOX6 is downregulated, suggesting an anti-proliferative role. We identified SOCS3 (suppressor of cytokine signaling-3) as an early SOX6-activated target. SOCS3 is a negative regulator of the cellular response to several pathways, including the EPO/Jak/STAT axis. In human erythroleukemic K562 cells, the overexpression of *SOCS3* recapitulates the block in proliferation elicited by SOX6. However, HEL cells (a different erythroleukemic cell line), which are made unresponsive to SOCS3 inhibition by the JAK2V617F+ mutation, keep growing until they complete their differentiation program [154]. Instead, in cells devoid of erythroid potential, such as in Sup-B15 B cells, the anti-proliferative role SOX6 can be achieved via the induction of apoptosis [155].

## 4. Splicing Factors in MDS and AML

**Figure 2:**
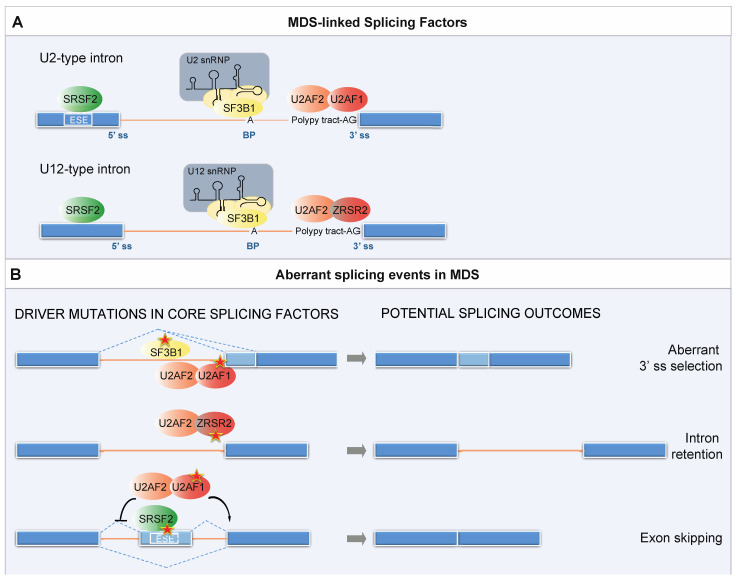
Splicing factors mutations in MDS. (**A**) Physiological role of MDS-linked SFs in spliceosome assembly. Intron removal requires the recognition of regulatory RNA sequence motifs by core components of the spliceosome followed by cleavage at conserved sequences called splice sites at the 5′ and 3′ ends of the introns (5′ ss and 3′ ss). Another important sequence is located 18–40 nucleotides upstream from the 3′ end of an intron. This sequence, termed the branch point (BP), always contains an adenine, but it is otherwise loosely conserved. Splice site recognition additionally requires Exonic Splicing Enhancers (ESEs). ESEs participate in both alternative and constitutive splicing, and many of them act as binding sites for members of the SR protein family, among which SRSF2. U1, U2, U4/U6, and U5 snRNPs are fundamental for the recognition of the splice sites and in the catalysis of the splicing reaction. Each of the five snRNPs contains one small nuclear RNA (snRNA) and a number of protein components. The initial intron recognition is achieved by U1 snRNP, which binds to the 5′ ss through base-pairing between the 5′ ss and the 5′ end of U1 snRNA, and by the interaction of splicing factor 1 (SF1/mBBP) with the BP; the 3′ ss is bound by the U2 auxiliary factor U2AF (U2AF1/2 heterodimer). Subsequently, SF1 is displaced and U2AF recruits the U2 snRNP, in which the multi-protein component SF3b harbors the subunit SF3B1, to the BP sequence, forming the pre-spliceosome. The pre-spliceosome then associates with the preassembled U4/U6.U5 tri-snRNP and after several rearrangements gives rise to the catalytically active spliceosome. In addition to the major U2-dependent spliceosome, most metazoans contain a second distinct pre-mRNA splicing machinery called the minor (U12-dependent) spliceosome. U12-type introns account for less than 0.5% of all introns in any given genome and are enriched in specific gene families, such as the mitogen activated-protein kinase, voltage-gated sodium and calcium ion channels, and E2F transcription factors [160]. ZRSR2 is a U2AF1-related protein that interacts with U2AF2 and is required for the recognition of the minor introns’ 3′ ss. (**B**) Aberrant alternative splicing events caused by the MDS-linked SFs’ mutations. Mutations in different SFs result in distinct mechanistic alterations in splicing. *SF3B1* and *U2AF1* mutations mainly alter 3′ ss selection through recognition of a cryptic 3′ splice site, and enhance aberrant exon inclusion through the selection of non-canonical BPs. Aberrant splicing caused by *ZRSR2* mutations primarily consist in intron retention. Finally, *SRSF2* mutations alter its ability to recognize a splicing enhancer motif, resulting in the majority of cases in exon skipping.

Intron splicing is catalyzed by a large molecular machine termed the spliceosome, composed of five small ribonucleoprotein particles (snRNPs) and >100 proteins (Figure 2 and Box 1). In MDS, somatic mutations in the core components of the spliceosome were first identified by next-generation sequencing in 2011 [156,157,158]. Splicing factor mutations are very frequent in MDS, where mutations in *SF3B1, U2AF1, ZRSR2,* and *SRSF2* account for >50% of the cases [159]. Given the fundamental role of the splicing machinery, the mutations are almost always mutually exclusive heterozygous missense mutations.

BOX 1 **Splicing factors most frequently mutated in MDS.**

SF3B1 is the 155 kDa subunit of the SF3b complex, a multi-protein component of the U2 snRNP. Along with SF3B3 and PHF5A, it binds to the BP. Once the U2 snRNP is stably integrated into the pre-spliceosome, the U2 snRNA pairs with the BP region of the pre-mRNA to form the branch helix encapsulated within the SF3b protein SF3B1 [161]. The BP adenosine base is flipped out from the branch helix and interacts with SF3B1. The most common splicing abnormality caused by SF3B1 mutations is the usage of cryptic 3′ ss with a short polypyrimidine tract that are usually located −15 to −24 nt from the canonical 3′ ss [162].

U2AF1 is the 35 kDa subunit of the U2AF heterodimer. Its partner, U2AF2 (also known as U2AF65), binds to the polypyrimidine tract that precedes the conserved AG dinucleotide of the 3′ ss. U2AF1 interacts with the 3′ side of the boundary sequence between the exon and intron conserved and recognizes the AG dinucleotide. U2AF, like SF3B1, is associated with the U2 snRNP and promotes the assembly of the pre-catalytic spliceosome. Mutations in U2AF1 alter the 3′ ss selection and enhance aberrant exon inclusion. The crystal structure of U2AF1 in complex with a 3′ ss RNA shows that the ZFs also contribute to the preference for bases flanking the AG nucleotide, thus explaining how pathogenic mutations affect sequence specificity [163].

ZRSR2 can interact with U2AF2 [164] and with other components of the pre-spliceosome assembly, including SRSF2. ZRSR2 plays a role in pre-mRNA splicing of both the U2- and U12-type introns by contacting the 3′ ss. However, it facilitates different steps. While in the U2-type introns ZRSR2 is required for the second catalytic step, for the U12-type introns, it promotes formation of the pre-spliceosome [165]. Aberrant splicing primarily consists of intron retention [166].

SRSF2 has been shown to bind exonic pre-mRNA at specific motifs called a splicing enhancer, where it acts as a splicing activator. Splicing enhancers are conserved nucleotide sequences, specifically recognized by SR proteins. These proteins bind to the specific intronic/exonic splicing enhancers via RNA recognition motifs and interact with other splicing factors, such as snRNP proteins. A recent solution structure of SRSF2 in complex with RNA revealed that SRSF2 has a consensus motif of SSNG (where ‘‘S’’ represents C or G) [167].

### 4.1. Splicing Factors’ Mutations in MDS and AML and Their Impact on Gene Expression

#### 4.1.1. SF3B1 (Splicing Factor 3b Subunit 1)

SF3B1 is the largest protein in the SF3b complex, with a central and C-terminal portion containing 20 tandem repeats termed the HEAT domain (Figure 2A). The HEAT domain of SF3B1 folds in a superhelical structure that is the central scaffold within the SF3b complex. In addition, SF3B1 harbors a stretch of U2AF ligand motifs (ULMs) at its N-terminus, which can specifically interact with the U2AF homology motif (UHM).

In myeloid malignancies, the frequency of *SF3B1* mutations is highest in MDS and in particular in MDS with ring sideroblasts (MDS-RS) [168]. According to the revised WHO classification a diagnosis of MDS-RS requires a somatic mutation in *SF3B1* and as few as (at least) 5% ring sideroblasts of nucleated red cells [169,170]. Many of the cancer-linked mutations occur in residues that are involved in the tertiary structure of the HEAT domain [161,171]. These substitutions likely affect SF3B1 conformation, possibly diminishing the interaction with the pre-mRNA and other spliceosomal proteins, thereby altering the selection of the 3’ss. Indeed, the K700E substitution, which accounts for the majority of *SF3B1* mutations among hematologic malignancies, promotes the use of cryptic 3′ splice sites through the selection of non-canonical branch points (BP, Figure 2B). In fact, by using computational and in vitro experimental approaches, Darman and colleagues demonstrated that the U2 snRNP still interacts with mutant SF3B1 but binds to a BP that is different from the one used by the wild-type U2 complex [162].

Heterozygous knock-in mouse models of *Sf3b1*K700E present impaired erythropoiesis, progressive macrocytic anemia without ringed sideroblasts, reduced HSC numbers, and host-repopulating fitness, as well as long-term hematopoietic stem cell (LT-HSC) expansion [172,173]. In both studies, enrichment analysis of the differentially expressed genes revealed significant changes in genes involved in RNA processing and metabolism but found little overlap between aberrantly spliced mRNAs in mouse versus *SF3B1*-mutant MDS patients’ cells [172,173], likely due to the poor conservation of intronic DNA sequences between species.

Interestingly, SF3B1 participates also in the minor U12-dependent spliceosome (Figure 2A, [174]). However, the impact of hotspot *SF3B1* mutations on minor intron splicing has not been investigated yet.

#### 4.1.2. U2AF1 (U2 Small Nuclear RNA Auxiliary Factor 1)

U2AF1 is the small subunit of the U2AF complex (Figure 2A). The protein contains a central non-canonical RNA-recognition motif called the U2AF homology motif (UHM), involved in the interaction with U2AF2, flanked by two Zn-finger motifs (ZFs) and a C-terminal arginine/serine-rich (RS) domain. Models of the U2AF1–splice site RNA complex and complementary RNA-binding experiments suggest that the both ZFs play a cooperative role in the recognition of the conserved AG dinucleotide of the 3′ ss, whereas only the second ZF contributes to the interaction with U2AF2 [163,175].

The *U2AF1* gene is mutated in 5% to 10% of MDS cases [168]. *U2AF1* mutations are generally associated with an adverse prognosis and an increased risk of progression to AML. Most *U2AF1* mutations occur within the two ZF domains of U2AF1, with S34 (S34F and S34Y) and Q157 (Q157R and Q157P), located near the RNA-binding interface, being the most commonly mutated residues in patients with de novo MDS [175,176,177,178]. Using genome-wide analysis, independent studies recently reported that the S34F/Y mutations of U2AF1 alters 3’ss selection and enhances aberrant exon inclusion (Figure 2B), leading to hematological malignancies, including MDS [176,177]. The first crystal structures of wild-type U2AF1 and of the S34F/Y mutant protein in complex with a 3′ ss RNA show that the ZFs also contribute to the preference for bases flanking the AG nucleotide and that pathogenic *U2AF1* mutations affect sequence specificity [163]. Using single-molecule Forster resonance energy transfer (smFRET), Warnasooriya and colleagues determined the influence of wild-type or S34F-substituted U2AF1 on the conformational dynamics of U2AF2 [179]. In agreement with previous work [180], the authors show that the U2AF1 ZF influences the conformation of the RNA-recognition motif of U2AF2. The S34F mutation can favor either an open or a closed conformation, depending on the sequence of the bound RNA, thereby fine-tuning spliceosome assembly [179].

#### 4.1.3. ZRSR2 (Zinc Finger CCCH-Type, RNA Binding Motif and Serine/Arginine Rich 2)

*ZRSR2* is located on the X chromosome and encodes for a U2AF1-related protein. With U2AF1 it shares a common core, consisting of an UHM domain and two flanking ZF domains, but it differs at the N-and/or C-termini.

*ZRSR2* mutations are observed in ~5% of MDS patients, with a prevalence in MDS subtypes without ring sideroblasts and chronic myelomonocytic leukemia, and are associated with an elevated percentage of bone marrow blasts and higher rate of progression to AML [181,182]. In contrast to SF3B1, SRSF2, and *U2AF1*, *ZRSR2* mutations occur across the entire length of the gene [168].

Madan and coauthors investigated the consequences of *ZRSR2* mutations by RNAseq of MDS bone marrow of eight male patients [166]. The analysis revealed that all the genes harboring U12-type introns expressed at sufficient levels were mis-spliced. Aberrant splicing primarily consisted of intron retention (Figure 2B), although also several loci were identified that displayed aberrant usage of cryptic U2-type splice sites, resulting in the partial retention of the U12-type intron. Gene Ontology (GO) analysis of the mis-spliced gene dataset identified several genes that participate in either hematopoietic differentiation or are implicated in myeloid malignancies, such as members of the E2F transcription factors, regulators of MAPK signaling, and the tumor-suppressor gene PTEN [166].

Very recently, to investigate the impact of ZRSR2 mutations on minor intron splicing, Inoue and colleagues created a mouse model through the conditional Cre-mediated excision of exon 4 of *Zrsr2*, resulting in an early frameshift [183]. In this model, *Zrsr2* loss leads to a global impairment of U12-type intron splicing with over one-third of U12-type introns exhibiting significantly increased retention. Interestingly, *Zrsr2*-null HSCs show enhanced self-renewal and MDS [183].

#### 4.1.4. SRSF2 (Serine/Arginine-Rich Splicing Factor 2)

*SRSF2* is a gene that codes for an auxiliary splicing factor that belongs to the serine/arginine (SR) protein family of splicing regulatory proteins. *SRSF2* is mutated in ~5% to 15% of MDS cases [168] and *SRSF2* mutations are consistently associated with adverse MDS and AML outcomes [13]. Hotspot mutations at codon P95 have been reported [166,168,184,185].

Two elegant studies by Zhang and colleagues and Kim and coauthors showed that SRSF2 P95 hotspot mutations alter its ability to recognize a splicing enhancer motif, increasing the affinity of the mutant protein for the nucleotide sequence CCNG relative to the sequence GGNG (Figure 2). This change in sequence affinity results in genome-wide alternative splicing changes with a prevalence of exon skipping events [186,187,188] (Figure 2B). Moreover, the in vivo comparison of hematopoietic specific *Srsf2* heterozygous or knockout mice with P95H/wild-type mice revealed that the *Srsf2*P95H mutation induces MDS, a phenotype different from *Srsf2* loss-of-function [186].

### 4.2. Common Themes in Alternative Splicing Alteration?

A relevant question is whether mutations in the different splicing factors may dysregulate the overlapping genes or pathways affecting stem/progenitor cells. To identify aberrantly spliced transcripts associated with *SF3B1*, *SRSF2*, and *U2AF1* mutations in MDS hematopoietic stem and progenitor cells, Pellagatti and colleagues performed RNA-seq on CD34^+^ cells from 82 patients with MDS and 8 healthy control individuals [188]. Consistent with previous studies, their results indicate that mutations in different SFs result in distinct mechanistic alterations in splicing (Figure 2) and affect different genes, although some overlap was observed, as shown by GO analysis. Interestingly, some genes recurrently mutated in MDS were aberrantly spliced in patients with *SF3B1* and *SRSF2* mutations (i.e., *STAG2*); others in patients carrying *SRSF2* or *U2AF1* mutations (i.e., *EZH2* [186] and *BCOR*). Similar results were also obtained by Shiozawa and coauthors who performed transcriptomic analyses of 265 bone marrow samples from myelodysplasia patients, 58% of which had mutations in one or more SFs, including *SF3B1*, *SRSF2*, *U2AF1*, and *ZRSR2* [189]. This study further confirmed that SFs’ mutations are associated with thousands of alternative splicing events.

In addition to the synthesis of aberrant protein isoforms, dysregulated alternative splicing events can trigger degradation of the mRNA via nonsense-mediated decay (NMD), a quality-control mechanism that eliminates transcripts harboring a premature stop codon. Indeed, several studies reported that a substantial fraction of the aberrant splicing events could activate NMD [162,188,190].

Despite these advances, several questions remain unanswered. Only a few abnormally spliced target genes have been identified and the functional consequences of the expression of the aberrant protein isoforms has not been fully elucidated. Importantly, splicing alterations were rarely found in well-known driver genes of myeloid neoplasms. Thus, it seems unlikely that the pathogenesis of SF-mutated myelodysplasia can be explained by a single mis-splicing event. A relative lack of alterations in established driver genes rather supports the previously proposed concept that multiple splicing alterations may cooperatively contribute to the pathogenesis of MDS [187].

## 5. Genome Maintenance Pathways in HSCs and Their Implications in MDS/AML

In adult organisms, HSCs reside in the bone marrow niche and, during homeostasis, are mainly maintained in a quiescent state [1]. In response to stress, injury, or infections, HSCs can undergo massive proliferation to effectively replenish all blood cell lineages. Yet, the functional fitness of HSCs has been shown to progressively decline upon aging [2,3].

Misfunction of old HSCs has been partially attributed to the accumulation of DNA damage. On one hand, DNA damage, when misrepaired, can promote genomic instability and accumulation of mutations, eventually contributing to transformation and cancer progression. On the other hand, DNA damage checkpoints can lead to apoptosis, senescence, or differentiation, causing stem cells attrition and eventually bone marrow failure [191,192]. It is well established that a proper DNA damage response (DDR) is crucial for HSCs maintenance. In mice, deficiency in components of key DDR pathways drives HSC functional exhaustion, as shown by their progressively reduced repopulation ability in bone marrow transplantation. These include loss of DNA-damage checkpoint proteins (e.g., *Atm* and *Atr)* and defective DNA repair due to mutations in the homologous recombination (HR) (e.g., *Brca1, Brca2, Fancc,* and *Fancd2*), nonhomologous end-joining (NHEJ) (DNA-dependent protein kinase *DNA-PKcs*, *Ku80,* and *Lig4*), mismatch repair (MMR) (*Msh2*), nucleotide excision repair (NER) (*Ercc1* and *Xpd*), and components of the ubiquitin-dependent DDR pathway [193,194,195,196,197]. Bone marrow failure and high incidence of hematological neoplasms in patients with severe DDR disorders, including ataxia telangiectasia (carrying mutations in the *ATM* gene) and Fanconi anemia, emphasize the critical importance of DDR in hematopoiesis [193,198].

Earlier studies detected increased DNA damage markers, such as phosphorylated histone H2AX (γH2AX) in mouse and human HSCs [199,200], and comet assays measured actual accrual of DNA breaks in aged human CD34^+^ HSCs, which was associated with the HSCs’ quiescent state [201]. The quiescent HSC-enriched fraction was also found less efficient in DNA double-strand breaks (DSBs) re-joining upon a low dose of ionizing radiation [202]. These data show that dormancy, although necessary for HSC maintenance, entails vulnerability to DNA lesions. In fact, deep sequencing studies of normal human HSPC DNA isolated from newborn, young, and elderly individuals have shown that long-lived self-renewing HSCs accumulate mutations [203,204]. In MDS/AML patients, while MDS/AML cells contain driver oncogenic mutations, hundreds of mutations are also found in healthy, not transformed HSPCs at diagnosis, most of which are probably non-pathogenic mutations that occurred before HSPCs acquired the initiating mutation, which are then ‘captured’ during clonal expansion. Such pre-leukemic cells survive treatment, can acquire additional mutations, and contribute to relapse of the disease [9,203,205,206].

HSC vulnerability to chromosomal abnormalities is due, in part, to the use of NHEJ, which is the predominant pathway for DSB repair in quiescent cells [207]. NHEJ is error-prone and misrepaired DSBs largely accounts for chromosomal translocations and oncogenic rearrangements in leukemia [208]. In addition to NHEJ, Osorio and colleagues recently showed that various endogenous mutational processes drive spontaneous accumulation of mutations in HSCs throughout life and estimated accrual of approximately 14 somatic mutations per year [204]. Notably, the majority of loss-of function mutations in *TET2* in AML originates from single-base substitutions [209] and most mutations in MDS, including mutations in *DNMT3A*, are C-to-T transitions at CpG, suggesting that they are due to age-related deamination of methylated cytosine [25].

Altogether, these studies demonstrated that long-lived self-renewing HSCs accumulate mutations in a cell-cycle independent and age-related manner, indicating that they represent a likely cell of origin for hematopoietic malignancies. Such susceptibility to mutation accumulation and genomic instability is a plausible basis for the development of pre-leukemic HSCs and disease progression, underscoring the importance of a mechanistic understanding of DDR processes in HSCs/LSCs.

DNA damage can originate in HSCs from both endogenous sources, including transcriptional, replicative, and oxidative stress, as well as from environmental or therapy related challenges [191,192,210,211], as exemplified by topoisomerase II treatment, which frequently induces balanced translocations involving the MLL (mixed lineage leukemia) gene on chromosome 11q23, associated with therapy-related AML [212,213]. Two novel emerging players in maintaining genomic stability, corrupted in LSCs, are the regulation of R-loops formation associated with mutations in splicing factors and the modulation of (DDR) ubiquitination pathways by deubiquitinating enzymes.

### 5.1. R-Loops as a Source of DNA Damage

**Figure 3:**
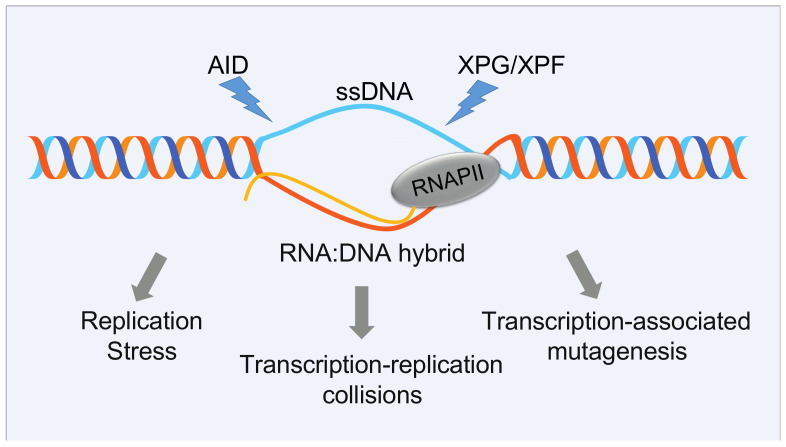
Detrimental consequences of unscheduled R-loop formation. Although R-loops are important for regulating diverse cellular events, they may have a negative impact on transcription elongation, DNA replication, and seriously compromise genome integrity, being a potential source of hypermutation, hyperrecombination, chromosomal rearrangements, or chromosome loss. The single-stranded DNA (ssDNA) that is exposed during R-loop formation is particularly vulnerable to DNA damage through the action of specific nucleases, such as activation-induced cytidine deaminase (AID), which promotes the conversion of dC to dU residues specifically on ssDNA, making the DNA susceptible to base excision repair enzymes, which in turn generate a DNA lesion, or XPF and XPG, two endonucleases involved in nucleotide excision repair. Alternatively, R loops may cause genome instability by stalling the progression of the replication fork. This may be the most relevant detrimental effect of R-loops in cycling S-G2 cells.

R-loops are three-stranded nucleic acid structures that form during transcription consisting of an RNA:DNA hybrid and a complementary displaced strand of DNA (Figure 3). While R-loops have regulatory roles in many physiological processes, unscheduled R-loops are a source of endogenous DNA damage since the displaced single-stranded non-template strand would be exposed to lesions or to processing by endonucleases. R-loop features have been extensively discussed in recent excellent reviews [214,215]. In addition, R-loops can interfere with DNA replication, leading to the stalling of the replication fork. Replication stress usually results in accumulation of stretches of exposed single-stranded DNA that can be processed to DNA double-strand breaks (DSBs). Thus, by impacting on DNA dynamics, R-loops constitute a threat to genome integrity [215,216,217] and, as such, they could exert selective pressure on preleukemic/CHIP clones and drive cancer progression [218,219,220].

#### 5.1.1. R-Loops and Mutations in Splicing Factors

Efficient splicing of intronic sequences should dramatically reduce the likelihood of R-loops formation [221]. Consistent with this hypothesis, one of the earliest reports of R-loop-induced genome instability was in cells lacking the splicing factor SRSF1 [222]. Later, siRNA screens revealed that the individual perturbation of various splicing factors leads to R-loop accumulation and DNA damage [221].

In the light of the role of pre-mRNA splicing and of SFs in preventing R-loop formation, it is not surprising that accumulation of R-loops, DNA replication stress, and activation of the ATR–CHK1 pathway were observed in *Srsf2*P95H mice and in HEK293T cells expressing the two most frequent SF mutations, *SRSF2* P95H and *U2AF1* Q157P [219]. Two recent studies further confirmed that R-loops accumulating in MDS cells critically depend on the ATR pathway for their survival. In the first, an *SF3B1* mutation in K562 cells and MDS CD34^+^ cells showed increased R-loop levels and enhanced activation of the ATR pathway [223]. In the second, similar results were observed in MOLM13 cells expressing *SRSF2* P95H and in CD34^+^ cells from *SRSF*2, *SF3B1*, and *ZRSR2* mutant MDS patients [218]. Both studies demonstrate that cells expressing SFs mutations are sensitive to inhibitors of the ATR/CHK1 pathway. However, the treatment with an ATR inhibitor of cells expressing mutant *SRSF2* induced further changes in alternative splicing, suggesting a note of caution in view of possible pharmacological ATR targeting [218].

In addition to directly inducing R-loops accumulation, SF mutations may alter genes controlling R-loops homeostasis, such as *SETX,* an RNA helicase that resolves R-loops [224] dysregulated in MDS patients carrying SFs mutations [188,218].

#### 5.1.2. Interplay between R-Loops and the Fanconi Anemia DNA Repair Pathway

Children with the inherited syndrome Fanconi anemia (FA) present hematopoietic abnormalities, including bone marrow failure and predisposition to MDS and leukemia, caused by HSC depletion and malignant transformation [38]. Mutations in any of at least 22 genes (*FANCA*–*FANCW*) that cooperate in the FA DNA repair pathway (also known as FA/BRCA) have been described in association with a Fanconi phenotype [198]. The FA/BRCA pathway plays a critical role in preserving genome integrity, with its canonical function being in the repair of DNA interstrand crosslinks (ICLs) through homologous recombination (HR) and with increasing roles in stabilizing replication forks, mitigating replication stress, and regulating cytokinesis [198].

Emerging evidence supports a strong link between the FA/HR genes and RNA:DNA hybrids. Molecular analysis by DNA:RNA immunoprecipitation techniques and cellular immunostaining with anti-DNA:RNA-specific monoclonal antibody S9.6 have shown that R-loops accumulate in different human and murine cell lines defective in FA proteins [225,226,227,228]. Notably, R-loops physiologically accrued also in primary myeloid Gr1+ and lymphoid B220+ committed bone marrow cells from FANCD2-deficient mice, implicating R-loops as a potential endogenous source of genomic instability in the FA phenotype. In support of this hypothesis, spontaneous DNA breaks accumulate in human cells depleted in FANCD2 in a manner dependent on co-transcriptional R-loops formation [216,225].

The FA pathway may have several means for R-loops control in distinct conditions. FANCD2 [198] is a key protein in the pathway, recruited to nuclear foci in a manner dependent from R-loops, likely by its ability to bind to RNA:DNA hybrids [227]. Moreover, FANCD2 was mostly found at very large transcribed genes and at highly transcribed loci, including Common Fragile Sites [225,228] and at sites of active transcription elongation [226,227]. Large fragile genes contain huge introns and require the splicing factor SFPQ for transcription. Along this reasoning, it has been proposed that FANC2 may promote R-loops resolution by recruiting and interacting with RNA processing factors such as hnRNPU and DDX47 helicase [229]. Of note, recent proteomic and molecular studies in HEK293T and U2OS cells identified a FANCI and FANCD2 functional association with SF3B1, frequently mutated in MDS [168], and pointed to a role for these FA proteins in regulating the splicing factor dynamics in chromatin as well as splicing outcomes [230]. It is not known if the FANCI or FANCD2 interaction with SF3B1 is relevant in malignant cells of FA patients.

The breast cancer susceptibility factors BRCA1 (FANCS) and BRCA2 (FANCD1) are key players in homologous recombination and act downstream of the FANCI/FANCD2 complex to repair the DSBs formed by ICLs processing [198]. Homozygous mutations in *BRCA1* and *BRCA2* genes are often hypomorphic, with residual activity to allow survival, and are associated with development of Fanconi anemia [231,232]. Consistently, both genes are required for proper HSC function and their haploinsufficiency confers to mouse HSPCs enhanced sensitivity to the crosslinking agent mitomycin C (MMC), a classical hallmark of FA [194,195,233]. Notably, children with biallelic mutations in the *BRCA2* develop AML in the first decade of life, sometimes with and sometimes without preceding MDS [121].

BRCA1 and BRCA2 have been recently involved in various ways in R-loops homeostasis [234]. R-loops accumulate globally at high levels in BRCA1- and in BRCA2-deficient cells [235,236,237], specifically at promoter proximal sites [238], driving DNA damage and chromosomal structural aberrations. Mechanistically, BRCA1 can directly interact with RNA:DNA hybrids and acts in concert with other factors, such as the DNA/RNA helicase SENATAXIN, to promote R-loops dissolution at the gene terminators [235]. Alternatively, BRCA1 cooperates with BRCA2, which in turn can recruit RNase H2 for R-loops dissolution at DSBs [239]. However, R-loops appear to be a double-edged sword. In fact, excessive amounts of R-loops may also negatively impact on the BRCA1 and BRCA2 function as exemplified in Ewing’s sarcoma patient cells. Here, the retention of BRCA1 at stalled transcriptional complexes associated with R-loops is thought to sequester the available BRCA1 protein, thereby impairing HR and repair [240]. Similar conditions of functional BRCA1 haploinsufficiency may occur in R-loops-driven MDS and may offer opportunities to therapeutic treatments, such as PARP inhibitors that sensitize BRCA1-deficient tumors [240].

HSCs are vulnerable to endogenous damage. In line with this, besides sensitivity to exogenous cross-linking agents, *BRCA2* mutant HSPCs display spontaneous chromosomal aberrations [233]. In particular, product intermediates of alcohol metabolism, such as formaldehyde and acetaldehyde, act as a potent endogenous source of ICLs in HSPCs and the FA pathway has a critical role in protecting HSC form aldehyde-induced DNA damage [241]. Notably, simple aldehydes can induce RNA:DNA hybrids formation in *FANCD2* deficient cells [226]. In addition, aldehydes impair BRCA2 function, ultimately inducing replication stress and chromosomal aberration via the unscheduled formation of RNA:DNA hybrids [237]. These findings suggest that exposure to aldehydes, both of environmental and/or endogenous origin, could act as source of R-loops-mediated DNA damage in HSPCs and potentiate spontaneous mutagenesis. This is more evident in vulnerable cells from carriers of mutations in *BRCA2* and other FA genes, but it can also contribute to malignant transformation in otherwise normal cells [237].

Finally, genomic instability and accumulation of unrepaired DNA lesions can activate innate immune and pro-inflammatory responses, which are crucial to hematopoiesis under physiological and stress conditions [242]. In particular, sensing of cytosolic DNA by the cyclic GMP-AMP synthase (cGAS)-stimulator of interferon genes (STING) induces a type I interferon response [243], which drives HSCs out of quiescence, leading to attrition and replicative stress [244]. Given the ability of RNA:DNA hybrids to bind and activate cGAS in vitro [245], accumulated R-loops could directly or indirectly, via induced DNA damage, contribute to activation of innate immune responses in normal and/or malignant HSCs [225,226,227,228,229]. In line with this, careful regulation of the R-loops levels might be critical for limiting the cGAS–STING inflammatory pathways and HSPC production, with a mechanism similar to that observed in *Ddx41* mutant zebrafish models during development [246]. In light of the potential of DNA damage-stimulation of immune responses in the context of cancer therapy [247], it will be important to further explore the connections between R-loops, DNA damage, and inflammation in HSPC and LSC biology.

### 5.2. Deubiquitinating Enzymes in HSCs Genome Stability

Post-translational modification by the 76 amino acids protein ubiquitin (ub) occurs via a step-wise process, involving the sequential activities of E1, E2, and E3 enzymes [248]. Reversal of ubiquitination is achieved by the activities of deubiquitinating enzymes (DUBs; also referred to as deubiquitylating enzymes or deubiquitinases), of which the human genome encodes approximately 100 members [249,250]. DUBs can cleave and edit ubiquitin modifications from substrate proteins, thereby critically modulating ubiquitin-mediated pathways, including protein homeostasis, cell cycle, genome maintenance, and epigenetic and receptor signaling [249,251]. Given the importance of ubiquitin modifications in controlling essential cellular processes, DUBs deregulation contributes to many human diseases [249,252].

**Figure 4:**
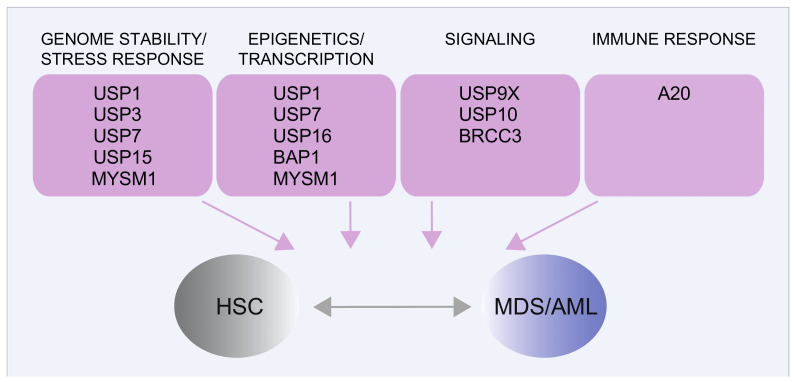
DUBs linked to normal HSC function and their implications in MDS/AML. The main DUBs involved in normal HSC or in MDS/AML biology and the main related cellular processes are shown. Additional functional roles of the same DUBs have been described in other cellular contexts and diseases [249,252,278,279].

It is well established that protein regulation via the ubiquitin–proteasome system is crucial to normal and leukemic HSC function [53,253]. Accordingly, in recent years, it has become clear that altering the function of DUB affects HSC homeostasis and may contribute to the onset of MDS and AML (Figure 4) by several mechanisms [254], as described for the DUBs BAP1, a tumor suppressor that cooperates with the polycomb group protein ASXL1 [255,256,257] or A20 [258,259], BRCC3 [260,261,262], USP7 [263,264], and USP10 [265]. DUB gene rearrangements have been reported in pediatric AML [266,267,268] and mis-splicing of *USP9X*—and its closely related protease *USP24*—was detected in *SRSF2* mutant MDS samples [269]. Finally, consistently with a crucial role of DDR in HSC self-renewal [193,199,270], an increasing number of DUBs impacting on DDR have been implicated in HSC maintenance [271]. These include USP1 [272], USP3 [196], USP15 [273], USP16 [274,275], and MYSM1 [276,277]. Here, we will focus on recent findings on DDR-related DUBs in myeloid malignancies and briefly discuss a few examples of emerging targeting opportunities of DUB enzymes.

USP1, in association with its cofactor UAF1, deubiquitinates three key DNA repair proteins: mono-ubiquitinated FANCD2, FANCI, and PCNA. First, through deubiquitination of FANCD2 and FANCI, USP1 acts as critical regulator of DNA crosslink repair by the Fanconi anemia pathway [280]. Consistently, loss of USP1 in mice confers sensitivity to ICL-inducing agents and impairs HSC function [272,281]. Second, through deubiquitination of PCNA, USP1 regulates the translesion synthesis pathway (TLS) [282]. This DNA damage tolerance pathway is required in mouse HSC for preventing replication stress, skewing of hematopoiesis towards myeloid/erythroid-biased progenitors and HSC failure [197]. Deubiquitination of PCNA by USP1 is also critical for replication fork stability in BRCA1-deficient cells. Indeed, inhibition of USP1-UAF1 by the small molecule ML323 [283] destabilizes replication forks and decreases the viability of the BRCA1-deficient cells, suggesting that USP1 inhibitors may have potential therapeutic use in BRCA1-deficient cancers [284]. Given the impact of USP1 on the FA pathway and on DNA damage tolerance, USP1 inhibition may also offer an opportunity for sensitizing FA/BRCA- and TLS-mutated MDS/AML cells.

In addition to DDR proteins, USP1 deubiquitinates a number of other substrates, including the inhibitor of DNA-binding protein 1 (ID1). ID1 is a helix–loop–helix transcription factor that regulates cell differentiation in various systems [285] and has been reported to cause a myeloproliferative disease in mice upon overexpression [286]. Different USP1 small-molecule inhibitors were reported to promote ID1 degradation and to induce cytotoxicity in leukemic cell lines [287]. However, although USP1/UAF1 represents a promising drug target, selective USP1 inhibitors require further pre-clinical assessment before determining their advancement into clinical development [288].

USP7 (or HAUSP) is one of the most studied DUBs due to its critical role in regulating TP53 function. As to the current view, USP7 inhibition destabilizes MDM2, the main E3 ligase targeting p53 for degradation, leading to p53 stabilization and p53-dependent tumor growth suppression [289,290]. Besides its action on p53, USP7 is largely involved in various cellular pathways, including genome stability maintenance, immune responses, epigenetic regulation, and HSC maintenance [291]. Within genome maintenance processes, USP7 acts as a master regulator of the response to DSBs through stabilization of the core components of DDR signaling, including the apical MRN–MDC1 complex, thereby impacting on both the NHEJ and HR DSB repair pathways [291,292]. Because of the important cellular roles of USP7, recent efforts focused on development of selective USP7 inhibitors as anticancer agents leading to the identification of several small molecules that are currently under pre-clinical testing [293]. Notably, toxicity of USP7 inhibitors can occur through mechanisms that generate DNA damage in a manner independently of p53, indicating that also p53-deficient cancer can respond to these compounds [294].

USP7 is involved in hematological malignancies and the USP7 small-molecule inhibitor P5091 impaired tumor growth in multiple myeloma xenograft models [295]. Implications in MDS and AML only recently emerged. In AML, Cartel and colleagues reported that the early USP7 inhibitor P22077 reduces viability of primary AML cells in vitro and tumor burden in vivo in PDX (patient-derived xenograft) models, and suggested USP7-mediated stabilization of CHK1 as one mechanism contributing to cytotoxicity [264]. In AML cells, high *USP7* expression correlates with chemoresistance. In line with this observation, the combination of USP7 inhibitors with chemotherapy increases toxicity in AML cell lines and in primary cells of patients with high USP7 levels, and could represent an option for the treatment of chemoresistant/relapsed AML [264]. Meanwhile, a proteomic study reported USP7 as a stable interactor of the Polycomb repressive complex PRC1.1. Notably, USP7 inhibitors strongly inhibited AML cell lines and primary AML cells proliferation in vitro, also independently of TP53 status, and significantly delayed leukemia development in a human MLL-AF9 xenograft mouse model [296].

Two other studies revealed a functional link between USP7 and MDS. In the first, chemical inhibition of USP7 reduced growth of CD34^+^ cells from MDS patients and MDS cell lines [263]. In the second, USP7 inhibitors as well as USP7 depletion cause a proteasome-dependent decrease in the GATA1 levels, impairing erythroid differentiation [297]. Indeed, USP7 can directly interact with GATA1 and remove polyubiquitin chains [297]. Given the role of GATA1 in erythropoiesis [118] and its altered expression in MDS [298,299], it will be interesting to further explore the mechanistic link between GATA1 and USP7 [297].

USP15, together with USP4 and USP11, form a closely related family of USPs, linked to DSB repair [273,300,301,302,303,304,305,306]. They are all expressed in early hematopoietic progenitors [196] and we recently scored them as hits in an in vivo shRNA screen for DUBs in mouse HSPCs, implicating these enzymes in HSC homeostasis [273]. Indeed, USP15 was validated as a critical DUB in HSC function and genome maintenance in *Usp15* knockout mice [273].

USP15 has been functionally implicated in various cancers [305,306,307,308], but only recent work uncovered its role in myeloid leukemia [273,309]. In our study, *USP15* was found overexpressed in human blood cancers, with the highest expression in AML. Furthermore, USP15 depletion impaired the viability of human CML and AML cell lines (M4-11, Kasumi-1) and increased their sensitivity to clastogens, including the crosslinking agent mitomycin C [273].

While USP15 regulates oncogenic pathways, such as TP53 [310,311] and TGF-b signaling [307], in AML cells we provided evidence that USP15 functionally interacts with FUS, stabilizing its protein levels [273]. As discussed above, FUS fusion proteins are involved in AML [76] and promote HSC self-renewal [312]. By modulating the FUS levels, USP15 might impact on FUS-dependent RNA processing [83], including R-loops accumulation, thereby accounting for the spontaneous DNA damage and micronuclei formation characteristic of USP15-deficient HSC and AML cells [273]. Alternatively, USP15 may influence the ability of FUS to promote efficient assembly of DNA repair condensates at DSBs sites [90]. Relevant to this point, USP15 was shown to be recruited to DSBs sites upon ionizing radiation [306]. In addition, USP15 may facilitate ubiquitin-dependent regulation of RNA splicing, as shown in HeLa cells [313]. Meanwhile, Niederkorn and colleagues identified USP15 in a protein complex with TIFAB in a del(5q) leukemia cell line (HL60). In this work, TIFAB, a factor implicated in MDS and AML with deletion of chromosome 5q, was reported to regulate USP15 deubiquitination activity and downstream p53 signaling [309].

Recently, Chen and colleagues identified as a major target of USP15 in melanoma models the DNA dioxygenase TET2, a driver mutated gene in MDS/AML [314,315].

The broad role of USP15 in regulating cellular processes [273,307,309,310,311,315,316], and the data reported above indicate that USP15 has context-dependent functions and involves multifaceted interactions. USP15-specific inhibitors have been recently developed by Teyra and colleagues [317] and represent a starting point to better understand USP15 function in normal and malignant hematopoiesis in vivo.

## 6. Emerging Therapeutic Targets

### 6.1. Transcription Factors’ Targeting

In principle, depending on the nature of the oncogenic mutation, several approaches for therapeutic targeting of TFs could be envisaged in order to hit the oncogenic protein, or to restore the correct level of expression of the wild-type protein. The final goal is to limit the expansion of HPSCs and to restore the proper differentiation pathways (“differentiation therapy”). The best proof of principle of the efficacy of this differentiation approach is demonstrated by the successful use of ATRA (all trans retinoic acid) plus arsenic-trioxide (ATO) in the treatment of promyelocytic leukemia caused by the PML/RARa translocation, which made this disease curable [318,319].

**Figure 5:**
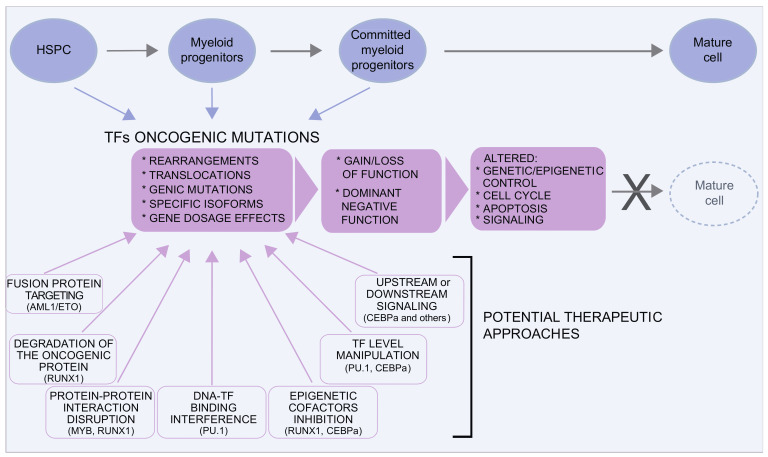
Transcription factors in MDS/AML. Several transcription factors are involved in MDS and AML. Oncogenic mutations are very heterogeneous and can result in gain or loss of function or in dominant negative effects, often with a pleiotropic effect on the control of the equilibrium between proliferation and differentiation. Although traditionally considered “undruggable” factors, in the last years, several approaches have been envisaged to either target the oncogenic protein or to restore the level/function of the TF. Some examples in this direction are discussed in the text and illustrated here.

However, targeting TFs is very difficult because of the absence of enzymatic activity and of evident druggable domains. Despite this, a variety of new targeting strategies are under development. They target the aberrant oncogenic proteins or their specific interactions, the recruited epigenetic cofactors, the TF–DNA interaction, or the TF expression level (Figure 5).

In the case of RUNX1, drugs that destabilize the RUNX1/ETO (t8;21) fusion protein by inhibiting its tetramerization [320] or activating miRNAs targeting the 3’UTR of *ETO* [321] are under development. More generally, the targeting chimera (PROTAC) approach aims to degrade the target protein by using molecules bridging unessential TF domains with ubiquitin ligases [322,323].

An alternative approach aims to target the specific interactions between the TF and relevant cofactors: Somerville and colleagues targeted the interaction of MYB with its TAF12 cofactor by overexpressing the TAF4 histone-fold fragment, thus suppressing MYB activity [324]. Oo and colleagues designed small drugs disrupting the binding of CBFβ-SMMHC (inv(16)) with RUNX1 [325].

Multiple evidence suggests that drugs targeting the dysregulated epigenome of AML cells can help to restore the control of the proliferation/differentiation balance (“epigenetic therapy” [326]. In this perspective, blocking the activity of aberrantly recruited epigenetic factors, would be very attractive. For example, because AML1/ETO recruits corepressors to RUNX1 targets, epigenetic drugs, such as HDAC inhibitors [327] or lysine-specific demethylase 1 (LSD1) inhibitors [328], demonstrated some efficacy. Interestingly, LSD1 inhibitors sensitize both AML and APL blasts to physiological concentrations of retinoic acid [329] and induce differentiation in AML cells by displacing the LSD1-coREST complex from the GFI1 enhancer, suggesting a wide potential of these drugs [330]. In the same line, pharmacological targeting of the MLL1 histone–methyltransferase complex recruited by the CEBPa p30 protein blocks proliferation and restores myeloid differentiation [331]. The major concern of these approaches is the specificity of the treatment, given the widespread pleiotropic role of the epigenetic factors. This problem could be limited by developing drugs targeting the specific interaction between the altered TF and its specific epigenetic cofactors.

TFs exert their action by binding to specific consensus sequences on DNA. Masking these sequences within the genome would thus avoid the binding of the oncogenic TF to its targets. Morita and colleagues moved on this direction and recently developed alkylating agent-conjugated pyrrole-imidazole (PI) polyamides that bind to the RUNX DNA consensus in order to prevent the binding of the RUNXs proteins (RUNX1, RUNX2, and RUNX3) to their targets in AML cells with intact p53. In these conditions, the loss of RUNX-binding and the pro-apoptotic p53 action induce cancer cell death [332].

Instead of blocking the accessibility of the DNA consensus sequence, Antony-Debré and colleagues developed a small molecule allosterically inhibiting the PU.1 DNA binding domain to reduce tumor burden [333]. This strategy sounds counterintuitive because a low PU.1 is frequent in AML. Moreover, PU.1 reduction is observed downstream to AML1/ETO and PML/RARa. Indeed, ATRA treatment of APL cells restores the PU.1 level, thereby resuming myeloid differentiation. However, the rationale for an approach based on blocking PU.1 binding is that a further reduction in PU.1 in already low PU.1 AML cells could induce their apoptosis. A similar approach could be envisaged in the case of GFI, where low levels of the protein but not the knock-out are associated with myeloid expansion [97].

In the case of reduced CEBPa, increasing the level/activity of this pro-differentiative TF could be a valuable option in promoting granulocytic differentiation. High-throughput screening identified small molecules selectively activating CEBPa [334] and more innovative strategies exploring short-activating RNAs (saRNAs [335]) are under development [336]. Other studies, beyond the main scope of this review, aim to restore CEBPa function by blocking the CEBPa upstream suppressor RAC1 [337] or by targeting the downstream CEBPa p30 tumor-promoting targets [338].

### 6.2. Targeting the Spliceosome

Hotspot SF mutations are invariably heterozygous, indicating that cells rely on the expression of the wild-type allele for survival. This observation suggests that mutant cells could have increased sensitivity to pharmacological perturbation of the spliceosome by splicing modulator drugs. In recent years several naturally derived and structurally distinct splicing inhibitors/modulators (e.g., pladienolides, herboxidienes, and spliceostatins, reviewed in [339]) have been identified. Many of these compounds target SF3B1 [340,341]. Notably, the K1071, R1074, and V1078 mutations confer resistance to these compounds [342]. Recent studies of the cryoEM structures of the SF3b complex bound to spliceosome modulators have shown that they primarily bind to the adenosine branch point binding pocket [161,343]. Artificial derivatives of these natural compounds have been developed that are currently under consideration in preclinical models of leukemia with SF mutations and in Phase I clinical studies. For example, Shirai and colleagues showed that *U2AF1*(*S34F*)-expressing hematopoietic cells are sensitive to sudemycin D6 [344], a small-molecule spliceosome inhibitor [345] that also targets SF3B1 [346]. Treatment of *U2af1(S34F)* transgenic mice with sudemycin results in an attenuation of mutant *U2af1*-induced hematopoietic progenitor cell expansion that is associated with increased cell death [347]. More recently, H3B-8800, a pladienolide-derived artificial small molecule that induces antitumor activity in xenograft leukemia models with core spliceosome mutations [171] has been used in a Phase I clinical trial (ClinicalTrials.gov Identifier: NCT02841540) in patients with MDS, AML, or in chronic myelomonocytic leukemia (CMML) [348,349]. In addition to SF3B inhibitors, very recent work has identified phenothiazine derivatives that inhibit early spliceosome assembly by specifically inhibiting the interaction of U2AF homology motifs (UHM) with U2AF ligand motifs (ULMs) [350]. These drugs could potentially interfere not only with the heterodimerization of the U2AF1 and U2AF2 but also with the activity of other splicing factors that contain UHM domains.

However, due to the fundamental role that pre-mRNA splicing plays in gene expression, there are concerns about the potential widespread toxicity of spliceosome inhibitors. Indeed, a Phase I clinical trial using E7107, a pladienolide derivative, against solid tumors was halted after two participants developed vision loss as a potential side effect of the drug [351].

### 6.3. Are DUBs Possible Therapeutic Targets?

Deubiquitinating enzymes are a relatively new class of drug targets. DUBs contribute to cancer through, at least in part, stabilization of the oncogenic proteins, modulation of the activity of the oncogenes or tumor suppressors proteins, or control of the epigenetic changes that promote tumor development [252]. Therefore, the generation of small-molecule inhibitors of DUBs is currently an active pursuit of the pharmacology industry [293,295]. Despite the intensive pre-clinical studies on selected DUBs, clinical translation of DUB inhibitors remains challenging and no inhibitor is currently being tested in active clinical trials [293,295].

A few examples of pharmacological DUB inhibitors inducing protein degradation for therapeutic benefit have been reported in hematologic malignancy models, including myeloid neoplasms [265,352,353,354] and multiple myeloma [353,355,356]. In AML, a recent study showed promising results for destabilization of the FLT3 mutant driver oncoprotein through inhibition of USP10. The FLT3 tyrosine kinase is a pharmacological validated target in AML, and *FLT3*-*ITD* (harboring internal kinase domain duplications) mutant expression is associated with poor prognosis. By screening a panel of DUB inhibitors, Buhrlage and colleagues [265] identified HBX19818 and P22077 as small molecules selectively promoting degradation of mutant FLT3 but not wild-type FLT3, and validated USP10 as the most potently inhibited DUB, over the previously described USP7 and other potential targets [265]. Interestingly, these USP10 inhibitors were efficacious in selectively suppressing the growth of mutant-*FLT3*-expressing cells versus cells expressing wild-type *FLT3*, including cells resistant to FLT3 kinase inhibitors, and their growth-inhibitory effect was validated in AML preclinical models, on primary patient tumor samples and patient-derived xenografts ex vivo [265]. The same groups reported that these compounds can also induce degradation of SYK and reduce SYK-driven leukemia cell proliferation [357]. SYK is a non-receptor tyrosine kinase involved in AML pathogenesis, highly activated in *FLT3*-*ITD^+^* AML and involved in the chromosomal translocation *TEL*-*SYK* in MDS [357]. Together, these results implicate USP10 inhibition as a potential therapeutic approach in patients harboring leukemic cells driven by oncogenic FLT3 or oncogenic SYK, or both, and could offer a strategy to override RTK-inhibitors resistance in this context [265,357]. In addition to the role of USP10, inhibition of USP9X by its inhibitors WP1130 or G9 is thought to preferentially induce apoptosis in AML cells harboring FLT3-ITD oncoprotein [358].

Overall, more than 40 DUB inhibitors were developed over the past decade. Although the majority still exhibit modest potency (micromolar range) and can target multiple DUBs, recent optimizations are moving the field forward [293,295]. These include X-ray crystallography of DUB-substrate/inhibitor interactions, DUB activity profiling [359], and development of ubiquitin variants with enhanced affinity for specific DUBs [317,360]. The recently developed USP7 inhibitors are the first promising examples of structure-based drug design, showing selectivity for USP7, nanomolar potency, and unique inhibitory mechanisms, through blocking ubiquitin binding by targeting either the active site or an allosteric site [293,295]. As mentioned above, the critical cellular roles of USP7 and the effects of first-generation USP7 inhibitors in MDS/AML cellular models [263,264,265] support further research. Promisingly, the novel and more specific USP7 inhibitor FT671 was highly effective in impairing the growth of primary AML cells [296].

An increasing number of papers implicates DUBs in regulating the fundamental processes in HSC maintenance [273] and the involvement of DUBs in MDS/AML has just emerged (Figure 4). A key, largely unresolved issue remains the identification of DUB substrates. Given the complex networks of functional interactions and the several targets of DUBs, it will be fundamental to identify their pharmacologically relevant substrates in specific leukemic cellular contexts [265,357].

The optimization of potent and selective DUB inhibitors for in vivo studies will help to assess the therapeutic potential of individual DUBs for the selection of effective drug targets and combinatorial strategies in MDS/AML patients.

## 7. Conclusions

The advent of extensive sequencing technologies has offered an unprecedented opportunity to gain insight into the complexity of the mutational landscape underlying myeloid neoplasms and their evolution, allowing to progressively define and refine molecular sub-groups.

In parallel with the expansion of sequencing technologies, the better understanding of the molecular mechanisms governing HSPCs biology and the lineage choice leading to the balanced production of mature cells has provided the rationale for the identification of novel potential targets/drugs for therapeutic interventions, as in the case of transcription factors, splicing factors, and deubiquitinating enzymes, the new key players in MDS and AML discussed in this review.

The paradigm of TFs being “undruggable” molecules is now challenged by a variety of targeting approaches. Small molecules inhibiting but also activating TFs are available. “Epigenetic therapy” directed against the chromatin modifiers recruited by TFs possesses great potential for wide application, but, for this same reason, raises serious specificity concerns. More recently, the direct targeting of specific protein–protein or DNA–TF interactions, by either masking the DNA-binding consensus on the DNA or by blocking the DNA-binding domain of the TF, have been proposed. Finally, the degradation of oncogenic TFs by the destabilization of their complexes, by miRNA-mediated degradation or by tagging them with ubiquitin, is becoming, in principle, an option. The targeted degradation approach could also be useful when a low level of a TF promotes cancer cell expansion but its further reduction induces cancer cell death.

The rational underlying modulation of pre-mRNA splicing in MDS carrying splicing factors’ mutations is to induce synthetic lethality. In this respect, small-molecule spliceosome inhibitors have demonstrated a high potential as novel therapies. However, since these drugs target the core components of the splicing apparatus, concerns about toxicity need to be addressed in pre-clinical and clinical trials. Nevertheless, inhibitors of additional splicing targets are in development and more therapeutic approaches will likely be determined. For example, R-loop formation, which is increased by splicing factors’ mutations, can sensitize cells to ATR inhibition.

In recent years, DUBs have been clearly confirmed to affect normal and malignant HSCs. Cellular and in vivo studies indicate that the functional and/or pharmacological inhibition of DUBs has therapeutic benefit in MDS/AML. Although the complexity of the DUB domains and the conservation of catalytic pockets pose challenges to the clinical development of small-molecule DUB inhibitors, recent X-ray crystallographic studies have demonstrated the possibility of designing selective and potent inhibitors with antitumor activity, as exemplified by the USP7 inhibitors that have therapeutic effects in AML in vivo models. The efficacy of co-application of DUB inhibitors with chemotherapy or kinase inhibitors, as well as their application to promote degradation of (onco) proteins with inherent or acquired resistance are valuable directions of research. As multiple DUBs contribute to HSC dysregulation in leukemogenesis, an in-depth understanding of their biological role and complex interactions in HSC biology and their perturbation in pre-malignant HSCs and LSCs is essential to the design of novel combinatorial treatments that affect the self-renewal of LSCs while sparing normal HSCs.

Large-scale population screenings have revealed that very often mutations found in AML cells are also present in non-malignant cells, in particular in elderly subjects. This observation poses the problem of evaluating the functional significance of this mutational heterogeneity in terms of the leukemic potential and functional relationship (cooperation, co-inhibition or neutrality) of co-occurring mutations.

In this scenario, molecular genetic screening at diagnosis, during follow-up for minimal residual disease monitoring, and at relapse will be crucial to trace the in vivo evolution of premalignant and malignant clones, with important implications for the prognosis and for the design of appropriate targeted therapies. Parallel functional approaches in cellular and patient-derived xenograft models will be complementary to assess the functional relevance of the mutations and to identify critical common mutations and/or pathways that could overcome the genetic heterogeneity and the mutational complexity of cancer cells. Hitting such common targets concomitantly with specific gene mutations through the design of combination treatments is a crucial issue for the development of effective therapies.

Although this new knowledge on key players and on the genetics and cellular aspects of leukemia onset and evolution have not yet reached full clinical translation, these studies will contribute to increase the repertoire of possible strategies to control, if not to eradicate, the disease through a personalized medicine approach.

## Data Availability

No new data were created or analyzed in this study. Data sharing is not applicable to this article.

## Abbreviations

AML: acute myeloid leukemia; BM: bone marrow; CH: clonal hematopoiesis; CHIP: clonal hematopoiesis of indeterminate potential; DDR: DNA damage response; DSB: DNA double-strand breaks; DUBs: deubiquitinating enzymes; FA: Fanconi anemia; HSC: hematopoietic stem cells; LSC: leukemic stem cells; MDS: myelodysplastic syndrome; SF: splicing factors; TF: transcription factors.
